# The 5BSL3.2 Functional RNA Domain Connects Distant Regions in the Hepatitis C Virus Genome

**DOI:** 10.3389/fmicb.2017.02093

**Published:** 2017-10-31

**Authors:** Cristina Romero-López, Alfredo Berzal-Herranz

**Affiliations:** Instituto de Parasitología y Biomedicina “López-Neyra”, Consejo Superior de Investigaciones Científicas (IPBLN-CSIC), Granada, Spain

**Keywords:** HCV, functional RNA domains, 5BSL3.2, HCV IRES, HCV CRE, long-distant RNA–RNA interactions

## Abstract

Viral genomes are complexly folded entities that carry all the information required for the infective cycle. The nucleotide sequence of the RNA virus genome encodes proteins and functional information contained in discrete, highly conserved structural units. These so-called functional RNA domains play essential roles in the progression of infection, which requires their preservation from one generation to the next. Numerous functional RNA domains exist in the genome of the hepatitis C virus (HCV). Among them, the 5BSL3.2 domain in the *cis*-acting replication element (CRE) at the 3′ end of the viral open reading frame has become of particular interest given its role in HCV RNA replication and as a regulator of viral protein synthesis. These functionalities are achieved via the establishment of a complex network of long-distance RNA–RNA contacts involving (at least as known to date) the highly conserved 3′X tail, the apical loop of domain IIId in the internal ribosome entry site, and/or the so-called Alt region upstream of the CRE. Changing contacts promotes the execution of different stages of the viral cycle. The 5BSL3.2 domain thus operates at the core of a system that governs the progression of HCV infection. This review summarizes our knowledge of the long-range RNA–RNA interaction network in the HCV genome, with special attention paid to the structural and functional consequences derived from the establishment of different contacts. The potential implications of such interactions in switching between the different stages of the viral cycle are discussed.

## Introduction

Predicting how RNA virus populations might evolve – a major public health goal – is a challenging task. RNA viruses, such as hepatitis C virus (HCV), replicate by virtue of a relatively low fidelity viral genome-encoded RNA-dependent RNA polymerase. It has been calculated that for each round of replication the mutation rate in HCV reaches a maximum of 1.3 nucleotides (nt) (Bartenschlager and Lohmann, 2000; Cuevas et al., 2009; Ribeiro et al., 2012). The variation introduced generates a dynamic genome pool composed of different but closely related sequences, referred to as a quasispecies (Martell et al., 1992). Quasispecies dynamics involve the constant sampling and selection of mutations that improve viral fitness. Such mutations are critical for escaping the host immune response, for drug resistance, the infection of new host species, and disease emergence. The acquisition of new mutations by viral genomes therefore provides a background for the action of natural selection. The magnitude of the effect of the acquired mutations is dependent on the environment: changes can be favorable for the infection in a given host but may have important fitness costs in a different environment, even within the same infected individual. This phenomenon creates a complex picture for the prediction of the disease severity and impedes the development of novel therapies (Manrubia and Lazaro, 2016; Perales and Domingo, 2016).

The balance between sequence promiscuity and functional conservation in viral genomes is made possible via the use of highly structured genomic regions that can absorb nucleotide variations – as long as these do not alter their active conformation. These are organized as discrete, in *cis*-functional RNA domains that operate in an interconnected manner to perform functions essential to the execution of the viral cycle (Romero-López and Berzal-Herranz, 2013). Such domains are considered information-carrying units beyond the nucleotide sequence. The study of the *cis*-acting functional elements, including their localization, sequence and structural conservation, is key to understanding viral infections at the molecular level. In addition, the essential role of genomic functional RNA domains in viral propagation and persistence makes them potential therapeutic targets.

Hepatitis C virus infection, which has a global prevalence of 2.8% (more than 185 million people are infected worldwide; Mohd Hanafiah et al., 2013), causes severe chronic disease that in many patients may eventually make liver transplantation necessary (Hoofnagle, 2002). Currently, the standard of care (SOC) involves the use of pegylated α-interferon combined with the modified nucleosides ribavirin and sofosbuvir (Kowdley et al., 2013; Lawitz et al., 2013). Unfortunately, interferon is not always well tolerated; in addition, the variability of the viral genome prevents a sustained therapeutic response. As mutants resistant to the current SOC arise, new targets and therapeutic agents must be ready. In this context, recent advances in the development of direct antiviral agents (DAAs) have prompted the progress of a new combined therapy consisting in the use of sofosbuvir plus the NS5A inhibitor velpatasvir and the NS3-NS4A inhibitor voxilaprevir (see below) (Bourliere et al., 2017). The two major benefits of this new strategy rely, on one hand, in the lack of α-interferon in this drug cocktail, which significantly reduces the secondary effects of the treatment; on the other, both velpatasvir and voxilaprevir operate as pangenotypic inhibitors, thus simplifying the customization of the therapy.

**FIGURE 1:**
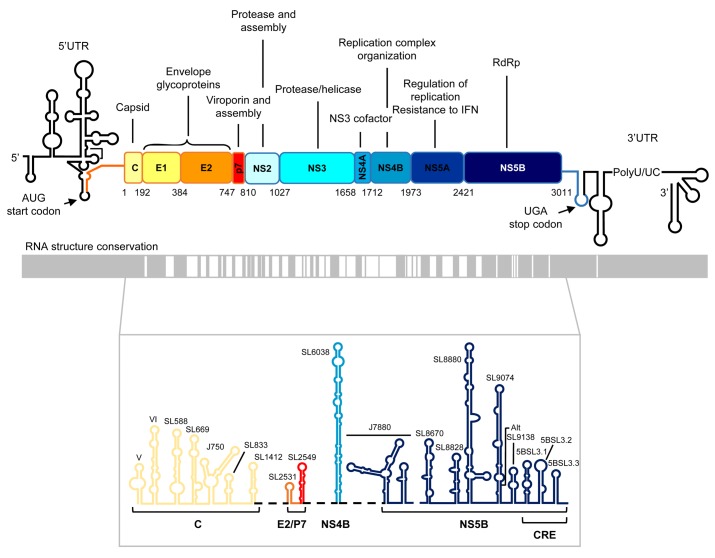
Genetic organization and functional RNA domains in the hepatitis C virus (HCV) genome. **Upper panel**: Diagram showing the genetic organization of the viral genome with the highly structured 5′ and 3′ untranslated regions flanking the single open reading frame (ORF). Viral structural and non-structural (NS) proteins and their functions are indicated. The translation start and stop codons are marked by arrows. Numbering corresponds to codon positions in the ORF according to the HCV Con1 isolate, genotype 1b. **Middle panel**: Structural conservation map of HCV RNA. Gray boxes denote those structurally conserved regions among different viral isolates. **Lower panel**: Conserved secondary structural elements in the ORF of the viral RNA genome. Stem-loop structures names are shown. CRE, *cis*-acting replication element, located at the 3′ end of the ORF. It consists of three highly conserved stem-loops, 5BSL3.1, 5BSL3.2, and 5BSL3.3. Color code and labels at the bottom indicate the position where each stem-loop is located.

Hepatitis C virus is a member of the family *Flaviviridae* and the genus *Hepacivirus*. Based on the phylogenetic analysis of genomic sequences, up to seven genotypes have been described for HCV showing more than 30% divergence at the nucleotide level. Closely related sets of subtypes and isolates have been identified as well (Smith et al., 2014). The viral genome is a positive RNA molecule of ∼9.6 kb that encodes a single ORF flanked by highly conserved untranslated regions (5′ and 3′UTR; **Figure 1**) (Choo et al., 1989). Viral proteins are produced as a single polypeptide that is co- and post-translationally processed by viral and cellular proteases to yield structural (core, E1, and E2) and non-structural proteins (p7, NS2, NS3, NS4A, NS4B, NS5A, and NS5B; **Figure 1**) (Grakoui et al., 1993). It is noteworthy that genetic variation does not occur evenly over the viral genome. On average, the complete sequence genome differs by about 31–33% per position (Smith et al., 2014), but only ∼10% of the entire viral genome is susceptible to positive selection (Patino-Galindo and Gonzalez-Candelas, 2017). The affected regions are intimately related to genotype prevalence (Messina et al., 2015). The 5′ end of the HCV genome and the viral capsid-encoding sequence are the most conserved regions, with around 80–90% sequence identity. The hypervariable region and the envelope protein-encoding sequence show the least sequence identity among isolates (**Figure 1**) (Le Guillou-Guillemette et al., 2007). These observations agree with the existence of highly conserved structural units throughout the HCV genome (Fricke et al., 2015; Mauger et al., 2015; Pirakitikulr et al., 2016). Some of these operate as essential functional domains – key targets for the development of new therapeutics and diagnostic agents. This review provides a brief overview of the *cis*-acting signals critical for HCV infection, with special emphasis on the core partner, i.e., the so-called 5BSL3.2 domain at the 3′ end of the viral ORF. It also focuses on the sequences and structural units of the viral genome essential to the preservation of the roles of 5BSL3.2 during the infective cycle.

## Functional RNA Domains in the HCV RNA Untranslated Regions

### Functional RNA Domains in the 5′ End of the HCV Genome

**FIGURE 2:**
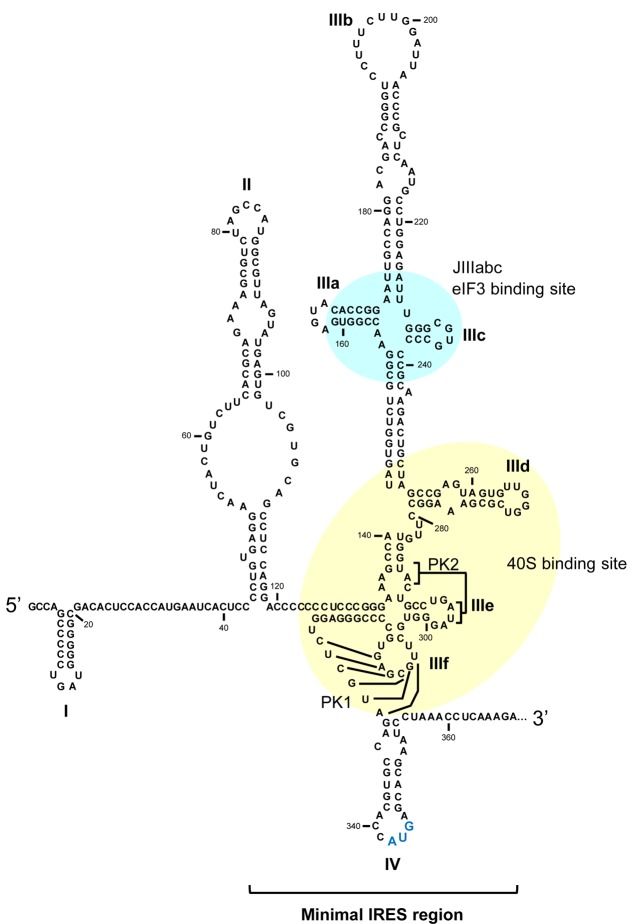
The HCV IRES region. Secondary structure of the 5′UTR in the HCV genome including the minimal internal ribosome entry site (IRES). Domains involved in the interaction with eIF3 and the 40S ribosomal subunit are marked in cyan and yellow, respectively. The translation start codon is shown in enlarged blue lettering. PK, pseudoknot. Numbering corresponds to nucleotide positions of HCV Con1 isolate, genotype 1b.

The HCV 5′UTR occupies the first 341 nucleotides of the HCV genome (**Figure 2**). This is one of the most conserved genomic regions, with ∼85% of sequence identity preserved across viral isolates (Bukh et al., 1992; Smith et al., 1995). Importantly, HCV protein synthesis and replication are governed by functional domains in the 5′UTR.

Viral translation is initiated by a mechanism different to the canonical cap-dependent pathway that dictates most cellular protein production. In HCV, an internal ribosome entry site (IRES), in the absence of any other factor, can direct the recruitment of the 40S ribosomal subunit (Tsukiyama-Kohara et al., 1992; Wang et al., 1993; Pestova et al., 1998; Lytle et al., 2002). The 48S particle is then constituted via interaction with the eIF3 and eIF2/GTP/Met-tRNA ternary complex (Sizova et al., 1998; Otto et al., 2002; Berry et al., 2011; Sun et al., 2013). The three-dimensional structure of the 48S complex allows for the proper positioning of the translation start codon in the P site (Berry et al., 2010, 2011; Quade et al., 2015; Angulo et al., 2016). Viral protein synthesis initiates after the binding of the 60S particle, plus the eIF5 and eIF5B factors, to the 48S complex, thus forming the productive 80S ribosomal complex (Yamamoto et al., 2014). This mechanism can be simplified by bypassing the recruitment of eIF3 and eIF2. In fact, eIF3-IRES binding is not essential for the initiation of viral translation *per se*. A recent publication has shown that this binding displaces the eIF3 from the canonical 43S pre-initiation translation complex, thus releasing the IRES binding site in the 40S ribosomal subunit and promoting the initiation of protein synthesis (Hashem et al., 2013). In addition, under certain stress conditions during which eIF2 is inactivated by phosphorylation, the HCV IRES can circumvent the eIF2-dependent delivery of the tRNA-Met by the GTPase-activating protein eIF5 (Terenin et al., 2008), or perhaps eIF2A (Kim et al., 2011) or 2D (Dmitriev et al., 2010). This strategy imitates the assembly of the translation machinery seen in prokaryotic cells.

The different proposed mechanisms provide important insights in the translation initiation pathway mediated by the HCV IRES (and other related viral and cellular IRESs). However, several questions remained unclear during past years. In a recent work (Jaafar et al., 2016), new roles for eIF1A were discovered, which may help to illustrate and complete the real stepwise pathway during HCV IRES-dependent translation initiation. The findings include the stabilization of the Met-tRNA binding to the IRES-40S pre-initiation complex, the help in the discrimination of improper AUG start codons and the stimulation of the eIF5B-dependent GTP hydrolysis. The authors proposed a revisited IRES-driven translation initiation model by which the HCV IRES would bind to a pre-assembled translation initiation complex, which is a remainder from the translation termination and ribosomal recycling events. This complex encompasses the 40S subunit, eIF3 and eIF1A. In the absence of the ternary complex eIF2/GTP/Met-tRNA, the IRES can easily dock to occupy the decoding groove, in good agreement with previous reports (Quade et al., 2015). In this step, the IRES can displace the eIF3 from its position in the 40S subunit (Hashem et al., 2013). The conformation of this new complex favors the recruitment of the Met-tRNA, in a process that should not necessarily require eIF2. This would be followed by GTP hydrolysis and the release of eIF2. Then, eIF1A and eIF5 could work together to confirm the use of the functional AUG codon, with the subsequent 60S ribosomal subunit binding. Both the model proposed by Jaafar et al. (2016) and the previous simplified version are supported by experimental data and can overlap to provide a wide view of the potential of the IRES to accomplish the complex task of translation initiation under adverse cellular conditions.

The extreme simplification of the initiation translation mechanism shown by the HCV IRES, compared to the canonical mechanism, is achieved through its complex, high-order structure, in which the canonical protein factors are substituted by functional RNA domains present in the viral genome. The minimal IRES region encompasses most of the 5′UTR and spans to nucleotide 372 within the coding sequence (**Figure 2**) (Reynolds et al., 1995; Honda et al., 1996b). Under physiological magnesium conditions, the IRES folds autonomously into three major domains – II, III, and IV – defined by simple or branched stem-loops (**Figure 2**) (Kieft et al., 1999; Perard et al., 2013). Far from being a rigid entity, the HCV IRES is an articulated region in which different domains move collectively to achieve the initiation of translation (Perard et al., 2013). Domains II and III appear aligned at both sides of a double pseudoknot motif (PK1 and PK2; **Figure 2**) (Berry et al., 2010), which guides the correct positioning of the translation start codon (domain IV) into the P site (Berry et al., 2011). Domain II is mostly involved in the constitution of the pre-initiation 48S complex and in inducing changes in the conformation of the 40S ribosomal subunit to promote the first round of ribosomal translocation (Spahn et al., 2001; Filbin and Kieft, 2011; Filbin et al., 2013; Yamamoto et al., 2015).

The highly branched domain III bears critical partners for the execution of viral protein synthesis. A collection of three- and four-way junctions organize different stem-loops (designated subdomains IIIa to IIIf; **Figure 2**), which operate as recruiting platforms for the binding of eIF3 (in the JIIIabc junction) (Lukavsky et al., 2000) or the 40S ribosomal subunit (mainly anchored in the critical subdomain IIId; **Figure 2**) (Jubin et al., 2000; Kolupaeva et al., 2000; Babaylova et al., 2009).

In addition to the well-known IRES-protein interactions, three GGG residues within the essential apical loop of subdomain IIId specifically recognize (via canonical Watson-Crick base-pairing) the highly conserved CCC triplet in structural element helix 26 of the rRNA 18S (Malygin et al., 2013a; Matsuda and Mauro, 2014). This interaction favors efficient and stable HCV IRES-40S binding, and seems to be specific for HCV RNA. Certainly, mutations in the rRNA 18S disrupting the IRES-40S contact do not affect cap-dependent translation (Matsuda and Mauro, 2014). Further, changes in the GGG triplet also block IRES translational activity (Jubin et al., 2000; Matsuda and Mauro, 2014; Angulo et al., 2016). A direct consequence of the interaction between IIId and 18S rRNA is a conformational rearrangement in the region surrounding the universally conserved nucleotide G1639 in rRNA, which unleashes the tRNA discrimination mechanism and the subsequent initiation of translation (Malygin et al., 2013a).

The molecular and functional organization of the HCV 5′UTR provides clear evidence of how information contained in genomic RNA domains governs the progress of the infective cycle.

### Structural and Functional Organization of the HCV Genomic RNA 3′UTR

**FIGURE 3:**
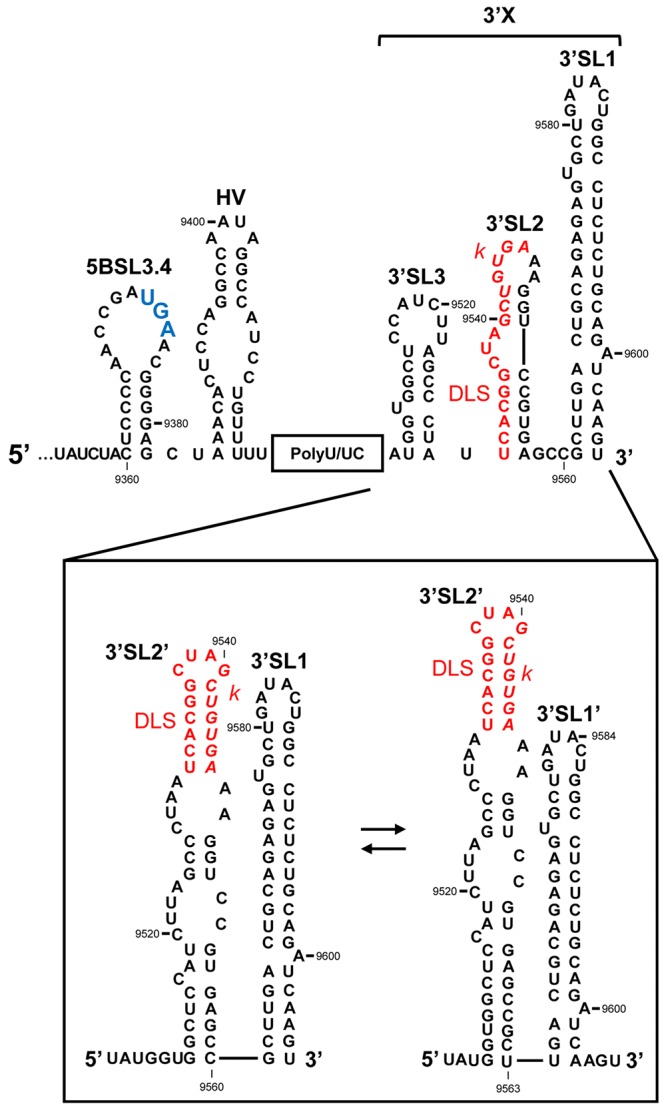
The 3′UTR of the HCV RNA genome. Secondary structure model proposed for the 3′UTR, showing the theoretical alternative conformations acquired in the 3′X tail. The palindromic motif involved in viral genome dimerization (DLS, dimer linkage sequence) is shown in red. The *k* sequence required for the interaction with the apical loop of the 5BSL3.2 domain is shown in italics. The translation stop codon is marked in enlarged blue text. Nucleotide numbering is as in **Figure 2**.

Further proof exists of the essential nature of genomic functional domains in HCV RNA. The HCV 3′UTR is a ∼200–250 nt-long sequence that contains essential functional domains required for viral replication and translational control (Kolykhalov et al., 1996; Fricke et al., 2015). Three well-defined regions are (**Figure 3**):

(i) The hypervariable region (HV); this is around 40 nts long and shows low-level sequence conservation among HCV isolates (Tanaka et al., 1995, 1996; Yamada et al., 1996). However, its secondary structure is preserved as a single stem-loop plus a 5′ region that partially overlaps the 5BSL3.4 domain (**Figure 3**) (Tanaka et al., 1996; Yamada et al., 1996; Ito and Lai, 1997). Though the hypervariable region seems to be dispensable for virus viability, its complete deletion leads to a significant reduction in HCV replication efficiency in cell culture (Kolykhalov et al., 2000; Friebe and Bartenschlager, 2002; Yi and Lemon, 2003).
(ii) The polyU/UC tract. This varies in length from one viral isolate to another, ranging from 30 to 80 nts (**Figure 3**; Kolykhalov et al., 1996). It consists of a homopolyuridine stretch interrupted by cytosine residues. Shortening the poly(U) tract below 27 nts, or interruption by CC dinucleotides, leads to the instability of the viral genome (Friebe and Bartenschlager, 2002; You and Rice, 2008). This suggests the recruitment to the polyU/UC tract of protein factors with special preferences for uridine over other pyrimidines. Interestingly, the HCV NS3 helicase, NS5A and NS5B (**Figure 1**) have been shown to preferentially bind poly(U) sequences *in vitro* (Gwack et al., 1996; Lohmann et al., 1997; Huang et al., 2005), which suggests a role for the poly(U/UC) tract in HCV replication.
(iii) The 3′X tail, located at the very 3′ end of the HCV RNA genome, is one of the most sequence- and structurally-conserved regions of the entire genome (**Figure 3**) (Kolykhalov et al., 1996; Blight and Rice, 1997). This 98 nt-long sequence is of major structural interest given its dynamic folding, which endorses the idea that the 3′X tail has an important regulatory function. Two mutually exclusive conformations (**Figure 3**) have been identified by chemical modification assays (Cristofari et al., 2004; Ivanyi-Nagy et al., 2006) and NMR (Cantero-Camacho and Gallego, 2015): a three stem-loop (3′SL3, 3′SL2, and 3′SL1) conformer (**Figure 3**, upper panel), which exposes the highly conserved *k* sequence motif complementary to the apical loop of the upstream 5BSL3.2 domain (see below) (Friebe et al., 2005), and a two stem-loop (3′SL2′ and 3′SL1/3′SL1′) conformer (**Figure 3**, lower panel). Nevertheless, recent NMR and SAXS studies have led to the proposal of the existence of a predominant, relatively rigid conformation defined by two stem-loops exposing the palindromic sequence motif DLS (dimer linkage sequence; **Figure 3**) (Ivanyi-Nagy et al., 2006; Shetty et al., 2010; Cantero-Camacho and Gallego, 2015; Cantero-Camacho et al., 2017). The DLS motif is involved in the formation of homodimeric genomes. In the two stem-loop conformation, a fine-tuned balance between two further isoforms is achieved, mediated by long-distance RNA–RNA interactions (see below). This conformational switch works via a slight gliding of 3 nts at the base of 3′SL1 (**Figure 3**) (Cantero-Camacho and Gallego, 2015; Cantero-Camacho et al., 2017) allowing the formation of 3′SL1′ (**Figure 3**). This conformation favors the initiation of primer-independent RNA synthesis via the viral RNA-dependent RNA polymerase NS5B protein (Kao et al., 2000). All this is consistent with the fact that a homodimeric genome operates as a preferential template for HCV polymerase (Masante et al., 2015), suggesting that the acquisition of the two stem-loop conformation is a pre-requisite for the progression of the infective cycle (Romero-López et al., 2014; Masante et al., 2015). Dimer genomic formation might also promote the generation of new recombinant variants during RNA synthesis (Morel et al., 2011; Scheel et al., 2013; Galli and Bukh, 2014), helping to improve viral fitness.

As well as its essential role in RNA replication, the 3′UTR functions as a translation enhancer (Mccaffrey et al., 2002; Bradrick et al., 2006; Song et al., 2006; Bung et al., 2010). This is mediated by the acquisition of a viral genome’s closed-loop topology, which resembles that adopted by mRNAs that are translated in a cap-dependent manner (Song et al., 2006; Weinlich et al., 2009; Bai et al., 2013). This conformation depends on the establishment of distant, direct long-range RNA–RNA contacts (Romero-López and Berzal-Herranz, 2009, 2012; Romero-López et al., 2012, 2014; Shetty et al., 2013). The interaction of the translational machinery with both ends of the viral genome is also critical in the viral genome achieving a circular isoform (Bai et al., 2013), which helps to retain the 40S ribosomal subunit during the translation termination step and favors ribosome recycling for the next round of protein synthesis (Bai et al., 2013).

The HCV 3′UTR thus plays various roles during the viral cycle, controlling the progress of infection via a collection of functional elements that can switch between different structural states.

## Conserved Structural and Functional Domains Within the Coding Sequence

In addition to RNA domains being located in the untranslated regions, numerous *cis*-acting signals have been identified in the ORFs of RNA viruses (for a review, see Romero-López and Berzal-Herranz, 2013). The search for unique, highly conserved structural and functional elements encoded in the HCV ORF has been going on for 15 years. The use of bioinformatic tools and secondary structure mapping, combined with genetic strategies, has finally provided a good overview of the global and local folding of HCV RNA (Tuplin et al., 2002, 2004; Mcmullan et al., 2007; Davis et al., 2008; Diviney et al., 2008; Chu et al., 2013; Fricke et al., 2015; Mauger et al., 2015; Pirakitikulr et al., 2016). The HCV genome is a highly compact molecule, with extensive base-pairing, helping to preserve viral RNA from cellular endonuclease-mediated degradation. Two of these degradation systems, both of which are involved in the innate immune response, include RNase L, which is specific for single-stranded regions, and the double-stranded-specific interference pathway (Li and Lemon, 2013). Importantly, by constraining the length of the helix segments, the viral genome has reached a perfect balance between single- and double-stranded regions with the aim of minimizing the effect of RNase L activity without unleashing the interference mechanism. This is consistent with the presence of alternate, extensive regions of compact folding throughout viral RNA genomes – the so-called GORS (genome-scale ordered RNA structure) elements. The existence of these elements correlates positively with host persistence and cell-to-cell movement (Simmonds et al., 2004; Davis et al., 2008; Witteveldt et al., 2014).

Up to 20 conserved RNA structural elements are scattered throughout the HCV ORF (**Figure 1**), expanding the functional repertoire of the viral genome via their participation in viral translation, replication, and infectivity (Tuplin et al., 2002, 2004; Mcmullan et al., 2007; Diviney et al., 2008; Chu et al., 2013; Fricke et al., 2015; Mauger et al., 2015; Pirakitikulr et al., 2016). Interestingly, the NS5B coding sequence is specifically enriched in functional RNA domains, with up to 10 distinct conserved stem-loop structural units able to drive HCV RNA and protein synthesis (**Figure 1**) (Smith and Simmonds, 1997; Walewski et al., 2001; Tuplin et al., 2002, 2004; Hofacker, 2004; Lee et al., 2004; You et al., 2004; Chu et al., 2013; Mauger et al., 2015; Pirakitikulr et al., 2016). One of the best-characterized functional RNA domains in the NS5B coding sequence is the so-called 5BSL3.2 domain (also known as SL9266 following the new standardized nomenclature system according to the genomic nucleotide position of the first 5′ paired residue of the stem-loop). The following sections focus on the molecular and functional features of this domain and its relationships with distant RNA elements in the HCV genome.

## The 5BSL3.2 Domain Maps Within the NS5B Coding Sequence

**FIGURE 4:**
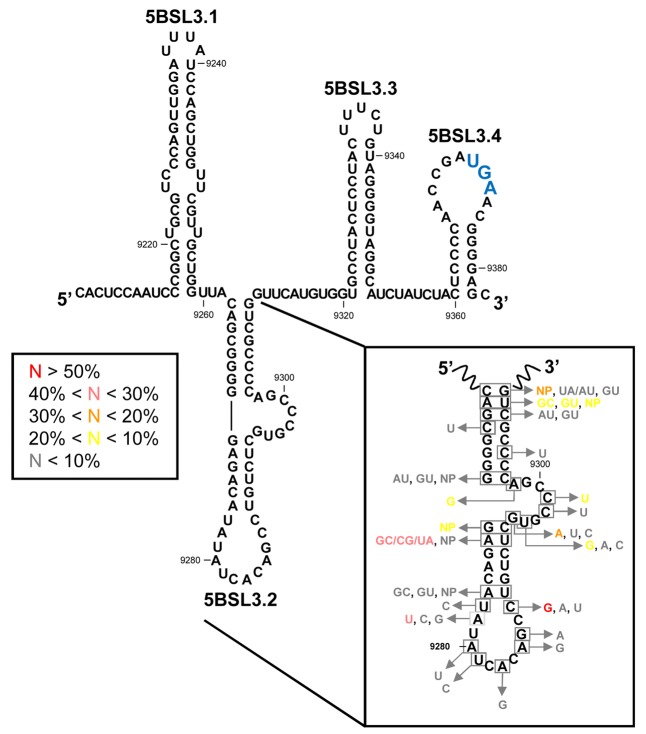
The CRE region. Sequence and secondary structure of the HCV CRE region, including the functional domains 5BSL3.1, 5BSL3.2, and 5BSL3.3 plus the 5BSL3.4 stem-loop. Conserved residues and covariant base pairs in the essential 5BSL3.2 domain, as described by You et al. (2004), are indicated in boxes. Colors denote the frequency for each nucleotide variation, as indicated. NP (non-paired) represents nucleotide variations impeding the formation of canonical base pairs. The translation stop codon is indicated in enlarged blue letters. Position numbering is as in **Figure 2**.

Using a combination of phylogenetic comparisons and thermodynamic prediction methods, Tuplin et al. (2002), defined the precise and conserved boundaries within the NS5B coding sequence that mark the limits of a set of genotypically well-conserved secondary structure elements. Five of these had already been proposed by other groups, either in whole or in part (Smith and Simmonds, 1997; Hofacker et al., 1998; Walewski et al., 2001; Tuplin et al., 2002). Nevertheless, it was in 2004 when three independent groups described the existence of a preserved sequence and structural region composed of three stem-loops in the 3′ terminus of the coding sequence. These were named 5BSL3.1, 5BSL3.2, and 5BSL3.3 (or SL9217, SL9266, and SL9324, respectively; **Figures 1**, **4**; Lee et al., 2004; You et al., 2004; Friebe et al., 2005). Reverse genetic analyses in hepatocytes bearing subgenomic replicon constructs, and in full-length viral replication models, showed these stem-loops to operate as a *cis*-acting replication elements (CRE). The central domain 5BSL3.2 was shown indispensable for HCV propagation (Lee et al., 2004; You et al., 2004; Friebe et al., 2005; Masante et al., 2015; Tuplin et al., 2015).

Bioinformatic prediction, biochemical structural mapping and NMR studies have all been undertaken to try to decipher the three-dimensional folding of 5BSL3.2 (Lee et al., 2004; You et al., 2004; Friebe et al., 2005; Tuplin et al., 2012), which was found to be a 48 nt-long imperfect hairpin with a 12 nt-long apical loop (**Figure 4**). The stem is interrupted at its 3′ end by an 8 nt-long bulge. Interestingly, both unpaired regions are phylogenetically conserved across different genotypes and show low synonymous site sequence variation (**Figure 4**) (You et al., 2004) that cannot be explained only by the need to preserve the NS5B coding sequence. Such a high degree of conservation undoubtedly points to a role in the functional control of the infective cycle, mediated by the apical loop and the bulge of the 5BSL3.2 domain (Lee et al., 2004; You et al., 2004; Friebe et al., 2005; Tuplin et al., 2012, 2015). Interestingly, the 5′-CACAGC-3′ sequence motif in the apical loop is found in other conserved elements of distantly related flaviviruses, such as Kunjin virus, West Nile virus or Dengue virus, where it operates as a single CRE (Markoff, 2003; Friebe et al., 2005). This observation points to the existence of common molecular mechanisms for viral RNA synthesis across different members of the family *Flaviviridae*.

Co-variation data confirm the existence in the basal part of the stem-loop of three out of the eight base pairs. In the upper stem, three of the six base pairs are invariable and two of the other remaining three can be predicted through compensatory base pair changes (**Figure 4**) (You et al., 2004). These phylogenetic data provide a convincing clue about the existence of the 5BSL3.2 hairpin *in vivo*.

**FIGURE 5:**
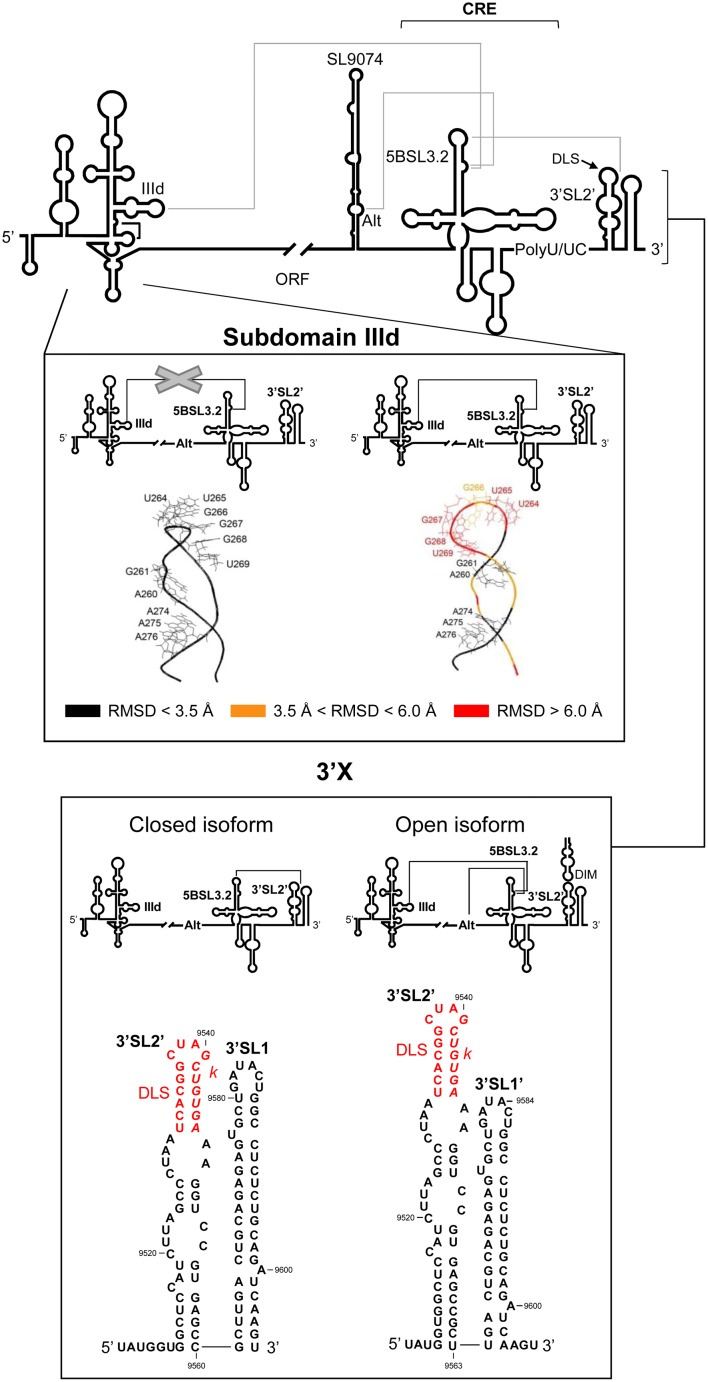
Long-range RNA–RNA interactions in the HCV genome promote conformational rearrangements in essential functional domains. **Upper panel**: Diagram of the secondary structure of the 5′ and 3′ ends of the HCV genome, including the functional domains involved in long-distance RNA–RNA contacts (solid gray lines). **Middle panel**: It shows the prediction for the structure of subdomain IIId (Romero-López and Berzal-Herranz, 2015). Root mean-square deviation (RMSD) values reflect differences in the conformation of subdomain IIId in the presence of the IIId-5BSL3.2 interaction (right) compared to its absence (left). Color code: Black, residues with an RMSD < 3.5 Å; orange, nucleotides with an RMSD ranging from 3.5 to 6.0 Å; red, residues with a RMSD of >6.0 Å. **Lower panel**: Structural reorganization of the 3′X tail depending on the establishment of different RNA–RNA contacts. The interaction of the 5BSL3.2 domain with the *k* motif in the 3′X region renders a two-stem-loop conformation in which the residues located at the 3′ end of the viral genome appear completely base-paired (left, closed isoform). However, contacts between 5BSL3.2 and upstream domains, such as the Alt sequence or subdomain IIId, release a three-nucleotide 3′ overhang in the 3′X tail (right, open isoform). DLS and *k* motifs are indicated according to **Figure 3**. Nucleotide numbering is as mentioned in **Figure 2**.

From a structural point of view, the molecular context surrounding the 5BSL3.2 domain is striking. It is embedded between two other stem-loops, 5BSL3.1 and 5BSL3.3, to yield a high-order structure that can be depicted as a cruciform element (**Figure 5**) (Lee et al., 2004; You et al., 2004; Friebe et al., 2005). Domains 5BSL3.1 and 5BSL3.3 were initially identified as evolutionarily conserved elements (Smith and Simmonds, 1997; Tuplin et al., 2004) that fold into stable stem-loop structures (**Figure 4**). RNase and chemical mapping have confirmed this secondary structure and support the proposed large cruciform structure (You et al., 2004). However, neither mutagenesis nor biophysical methods have yet confirmed the existence of this high-order structure *in vivo*. This might be due to the dynamic and relatively unstable nature of long-distance RNA–RNA contacts, which can promote the switch between different metastable structural states in the RNA molecule, depending on the presence of specific ligands or external stimuli. Thus, such contacts suggest a regulatory operation mode that might rely on the versatility and efficiency of the HCV CRE region.

## The 5BSL3.2 Domain Lies at the Core of a Complex RNA–RNA Interaction Network

The remarkable structural features of the 5BSL3.2 domain, as well as its strong conservation, point to its critical involvement in the HCV infective cycle. As mentioned above, the observation that both the apical loop and the bulge of the 5BSL3.2 domain are highly preserved among different genotypes and viral isolates suggests their participation in interactions with other viral RNA elements. Several reports have provided substantial proof of the existence of long-distance RNA–RNA contacts, together forming a complex network of interactions that appears to be governed by 5BSL3.2 (Friebe et al., 2005; Romero-López and Berzal-Herranz, 2009; Romero-López et al., 2012, 2014, 2017; Tuplin et al., 2012, 2015; Shetty et al., 2013; Fricke et al., 2015). Such a network would organize the three-dimensional folding of the viral genome into a compact conformation that correlates with virulence and the persistence of infection (Davis et al., 2008). To date, two conformational rearrangements of the HCV genome are known to be mediated by the 5BSL3.2 domain: viral genome circularization and the structural tuning of the 3′ end of the HCV genome.

### Viral Genome Circularization

Genome circularization is a crucial step in the initiation of viral protein and RNA synthesis in many positive-stranded RNA viruses (Villordo and Gamarnik, 2009). It depends on either direct, long-distance RNA–RNA interactions, or protein bridges that bring the ends of the viral RNA together. In most cases, a combination of both mechanisms is used. While no conclusive evidence exists for the acquisition of a closed-loop topology by the HCV genome, indirect data suggests it does indeed occur and depends on long-range RNA–RNA contacts (Romero-López and Berzal-Herranz, 2009; Romero-López et al., 2012, 2014; Shetty et al., 2013; Fricke et al., 2015). Using bioinformatic, biochemical, and biophysical methods, several independent groups have shown the establishment of a direct interaction involving the bulge of the 5BSL3.2 domain and the apical loop of subdomain IIId in the IRES element (**Figure 5**) (Romero-López and Berzal-Herranz, 2009; Shetty et al., 2013; Fricke et al., 2015). Importantly, this interaction occurs in the absence of protein factors. According to the proposed model, the essential nucleotide G263, located at the base of the apical loop of the subdomain IIId, would establish the initial contact with C9301 on the 3′ side of the bulge in the 5BSL3.2 domain (Romero-López and Berzal-Herranz, 2009). This contact theoretically extends along the unpaired regions of the participating domains. Nucleotide complementarity runs through the flanking stems up to A288 for the 5′ end, and to A9275 for the 3′ end (Romero-López and Berzal-Herranz, 2009). However, no such extended complex has ever been detected.

Conformational consequences derived from the interaction 5BSL3.2-IIId have been mapped by chemical probing coupled to three-dimensional structure prediction (**Figure 5**) (Romero-López et al., 2012; Romero-López and Berzal-Herranz, 2015). The aromatic rings of the residues located in the apical loop of subdomain IIId have been reported to change their orientation with respect to the solvent because of their interaction with 5BSL3.2 (**Figure 5**). Such conformational rearrangements would affect IRES function due to their interference with the efficient recruitment of the 40S ribosomal subunit mediated by the subdomain IIId (Malygin et al., 2013a,b). This is in good agreement with the idea that the 5BSL3.2 domain operates as a specific and efficient negative regulator during HCV IRES-dependent translation (Romero-López and Berzal-Herranz, 2012). The 5BSL3.2 is therefore considered a versatile multifunctional genomic element that takes part in different steps of the infective cycle and controls transitions between them.

### Structural Tuning of the 3′ End of the HCV Genome

The apical loop of the 5BSL3.2 domain establishes a long-range interaction with a complementary sequence, *k*, located in the apical loop of the downstream 3′SL2 element within the 3′X tail (**Figures 3**, **5**) (Friebe et al., 2005; Cantero-Camacho et al., 2017). The biochemical and structural properties of this interaction have been studied in depth following different biophysical and biochemical strategies (Friebe et al., 2005; You and Rice, 2008; Tuplin et al., 2012; Palau et al., 2013; Shetty et al., 2013; Cantero-Camacho et al., 2017). These analyses showed that the contact 5BSL3.2-3′X occurs in the absence of RNA chaperone proteins and is preferred over the formation of homodimeric viral genomes (Cantero-Camacho et al., 2017). Recent studies have determined that even for the dimerizable conformation of the 3′X region, which partially occludes *k*, the interaction with the 5BSL3.2 domain is made possible by the induction of the structural disruption of the two stem-loop conformations of the 3′X tail, thus unfolding the motif *k* (Cantero-Camacho et al., 2017). Hence, 5BSL3.2 may act as a structural cofactor promoting the acquisition of a new, functionally active folding of the HCV genome.

Conversely, the 5BSL3.2 bulge element may interact with the complementary sequence motif centered on position 9110 – the so-called Alt sequence (**Figure 5**) (Diviney et al., 2008; Tuplin et al., 2012; Shetty et al., 2013). This contact overlaps with that involving subdomain IIId. Both interactions show dissociation constant values in the same range, and seem equally likely to occur (Shetty et al., 2013). Choosing between them is affected by additional structural constraints and the presence of different cofactors (see below).

A significant feature of the interactions mediated by 5BSL3.2 at the 3′ end of the viral RNA is that they can be established in an independent and simultaneous manner (Tuplin et al., 2012; Palau et al., 2013; Shetty et al., 2013). This suggests that the binding sites are structurally independent. The connections established at the 3′ end render the formation of an intricate tertiary structure in which the 5BSL3.2 forms the core of an extended and dynamic pseudoknot (Tuplin et al., 2012). Reverse genetic and biochemical structural data have revealed this pseudoknot able to promote two alternative conformations in the 3′X region (Tuplin et al., 2012; Cantero-Camacho and Gallego, 2015; Kranawetter et al., 2017). In the open form, contact with the Alt sequence or with subdomain IIId would favor the establishment of the preferred two stem-loop conformation bearing three overhang nucleotides at the 3′ end of the HCV RNA (**Figures 3**, **5**). As mentioned above, this is a favored folding state for virus replication (Kao et al., 2000) and for genome dimerization (Palau et al., 2013; Romero-López et al., 2014; Cantero-Camacho et al., 2017). Alternatively, with the closed isoform, the interaction of 5BSL3.2 with the *k* motif in the 3′X tail would induce the local melting of the upper stem of 3′SL2′ and induce conformational rearrangements in the base of the 3′SL1′, leading to the formation of the extended stem-loop 3′SL1 (**Figures 3**, **5**) (Tuplin et al., 2012; Cantero-Camacho and Gallego, 2015). Interestingly, the thermodynamic equilibrium between these two isoforms seems to be dependent on the viral genotype, with the open conformation being favored by genotype 1b and the closed form by genotype 2a (Tuplin et al., 2012). These structural data undoubtedly suggest clear correlations between HCV RNA conformation, genotype-dependent virulence and even the viral sustained response to the treatment shown by the infected patient (Irshad et al., 2010; Carter et al., 2017).

The creation of this complex network of RNA–RNA interactions promotes the structural remodeling of the viral genome, not only in the directly involved domains, but also in distant regions. This phenomenon achieves complex and interconnected genomic-RNA-folding-dependent regulatory pathways. Indeed, the conformational rearrangement mediated by the 5BSL3.2 domain at the 3′ end is regulated by the IRES region, most likely as a consequence of the interaction IIId-5BSL3.2 (Romero-López and Berzal-Herranz, 2009; Romero-López et al., 2014). Using chemical structural mapping coupled to secondary structure prediction, it has been shown that the presence of both the IRES and the CRE regions favors the acquisition of the dimerizable two stem-loop conformation in the 3′X tail, exposing the DLS motif (Romero-López et al., 2014). This led to the assumption that the contact IIId-5BSL3.2 improves the dimerization of the viral genome. However, it has been recently reported that while the two stem-loop isoform is a requisite, it is not the only determinant affecting genomic dimer formation (Cantero-Camacho et al., 2017; Romero-López et al., 2017). In summary, the IRES and the CRE regions might be considered both cofactors and chaperoning agents that finely tune the three-dimensional folding of the 3′ end of the HCV RNA in order to control the different stages of the infective cycle, and the transitions between them.

The folding of the IRES region is also influenced by the presence of the 3′ end of the HCV genome. The 3′UTR and the CRE region have been shown to effect remarkable changes in subdomains IIIe and IIIf (Romero-López et al., 2012) – essential elements involved in the proper positioning of the translation start codon in the P site of the 40S ribosomal subunit (Berry et al., 2011). In addition, the three-dimensional organization of the eIF3 binding platform within the IRES can be modified by the interaction IIId-5BSL3.2, turning the typical S-turn conformation (Collier et al., 2002) into a more rigid form with altered functionality (Romero-López et al., 2012). Domain IV appears less stable in the presence of the 3′ end of the HCV RNA, which might contribute toward the proper positioning of the translation start codon (Honda et al., 1996a; Romero-López et al., 2012). All these findings are in good agreement with the acquisition of a circular topology by the HCV genomic RNA.

The 5BSL3.2 domain thus participates in the establishment of different long-range RNA–RNA interactions in the absence of proteins. This helps create a complex network of contacts, the careful regulation of which allows the HCV genome to acquire different functional conformational states, facilitating proper switching between the different stages of the viral cycle.

## Host and Viral Components Interact with the CRE

The recruitment of both host and viral components is important in the regulatory activities of the CRE region (Lourenco et al., 2008). The apical loop of the 5BSL3.2 domain binds to the viral polymerase NS5B protein (Zhang et al., 2005), favoring the positioning of the polymerase in the 3′X tail and thus the initiation of replication. The interaction of the NS5B protein with the apical loop of 5BSL3.2 might compete with the contact 5BSL3.2-3′X. Swapping from NS5B recruitment to 3′X interaction could therefore be used by the 5BSL3.2 domain as a regulatory mechanism for promoting transitions between steps during the infective cycle.

In recent years, exhaustive lists of cellular proteins susceptible to recruitment by the CRE region have been produced (Oakland et al., 2013; Ríos-Marco et al., 2016). Interestingly, some have been shown to influence viral translation, replication or both (Oakland et al., 2013; Ríos-Marco et al., 2016). Oakland et al. (2013) showed Ewing’s Sarcoma binding protein 1 (EWSR1) to interact with the CRE region; EWSR1 is a nuclear factor that regulates RNA synthesis and processing as well as the transport of pre-mRNAs involved in cell cycle progression and the response to DNA damage (Zinszner et al., 1994; Paronetto et al., 2011). It also binds other factors related to splicing, such as those belonging to the heterogeneous ribonuclear protein family (hnRNPs). Its regulatory role during mitosis has also been reported (Wang et al., 2016). The involvement of ESWR1 in tumorigenesis has attracted much attention since HCV infection can lead to the development of hepatocellular carcinoma. Though EWSR1 is preferentially located in the nucleus, it can be translocated to the cytosol of HCV-infected hepatocytes (Oakland et al., 2013). Here it binds to the viral genome in a manner dependent on the structure resulting from the interaction 5BSL3.2-3′X, promoting efficient HCV RNA replication, at least in cell culture (Oakland et al., 2013).

Subsequent proteomic analyses have identified a collection of RNA-binding proteins with highly conserved RNA recognition motifs able to bind to the CRE region (Ríos-Marco et al., 2016). hnRNPA1 is a very abundant nuclear and cytosolic protein involved in the packaging of pre-mRNAs into spliceosomal particles, and in the translocation of processed poly(A) mRNAs from the nucleus to the cytoplasm (Dreyfuss et al., 2002). During HCV infection, hnRNPA1 operates as an IRES-dependent translation initiation enhancer via its interaction with the IRES region (Lu et al., 2004), and as a negative regulatory partner of viral RNA synthesis, most likely by competing with NS5B for a common interacting site in the 5BSL3.2 domain (Ríos-Marco et al., 2016). Additionally, hnRNPA1 plays an important role as a splicing factor in the maturation of IFR3 (interferon regulatory factor 3), which is involved in interferon-mediated immunity (Guo et al., 2013). Sequestering hnRNPA1 by 5BSL3.2 would, therefore, influence interferon production, helping HCV escape the cellular immune response. HMGB1 (high mobility group box 1 protein) also interferes with HCV replication by binding to the CRE (Jung et al., 2011; Ríos-Marco et al., 2016), and is considered an antiviral factor (Jung et al., 2011). Finally, host proteins with helicase activity, such as DDX3, DDX5, and DDX17, might recognize the 5BSL3.2 domain and promote HCV replication and/or translation (Ríos-Marco et al., 2016). This last observation confirms the role of the CRE as a regulator of viral protein and RNA synthesis (Ariumi et al., 2007; Randall et al., 2007; Oakland et al., 2013; Ríos-Marco et al., 2016).

MicroRNAs (miRNAs) produced by the host cell have been identified as agents that regulate viral infection (Bruscella et al., 2017). Via a little-understood mechanism, miRNAs influence HCV infection at different stages. For example, miR-122, a highly abundant miRNA in hepatocytes, promotes viral translation and replication via its interaction with the IRES region at different target sites (Jopling et al., 2005; Jangra et al., 2010; Roberts et al., 2011), while miR-199a^∗^ represses RNA replication (Murakami et al., 2009). Let-7b also acts as a negative regulatory agent of HCV replication via its direct interaction with the region connecting domains 5BSL3.2 and 5BSL3.3 (Cheng et al., 2012). Let-7b is involved in cell differentiation and has been intimately associated with the development of cancer (Takamizawa et al., 2004; Yu et al., 2007). This, along with the observation that cell cycle regulatory proteins bind to the CRE, provides additional evidence of the potential role of the CRE region in HCV-associated hepatocellular carcinoma.

## The CRE Region is a Critical Switch Component in the HCV Infective Cycle

The HCV cycle is extensively compartmentalized, both spatially and temporally: viral protein synthesis, replication, and encapsidation occur in different cellular localizations and do not overlap in time (Shulla and Randall, 2015). The switch between one stage and the next must therefore be carefully controlled. The machinery required for this is not well-understood, but it is widely assumed that functional genomic domains, along with host and viral factors, together control the progression of the infective cycle.

**FIGURE 6:**
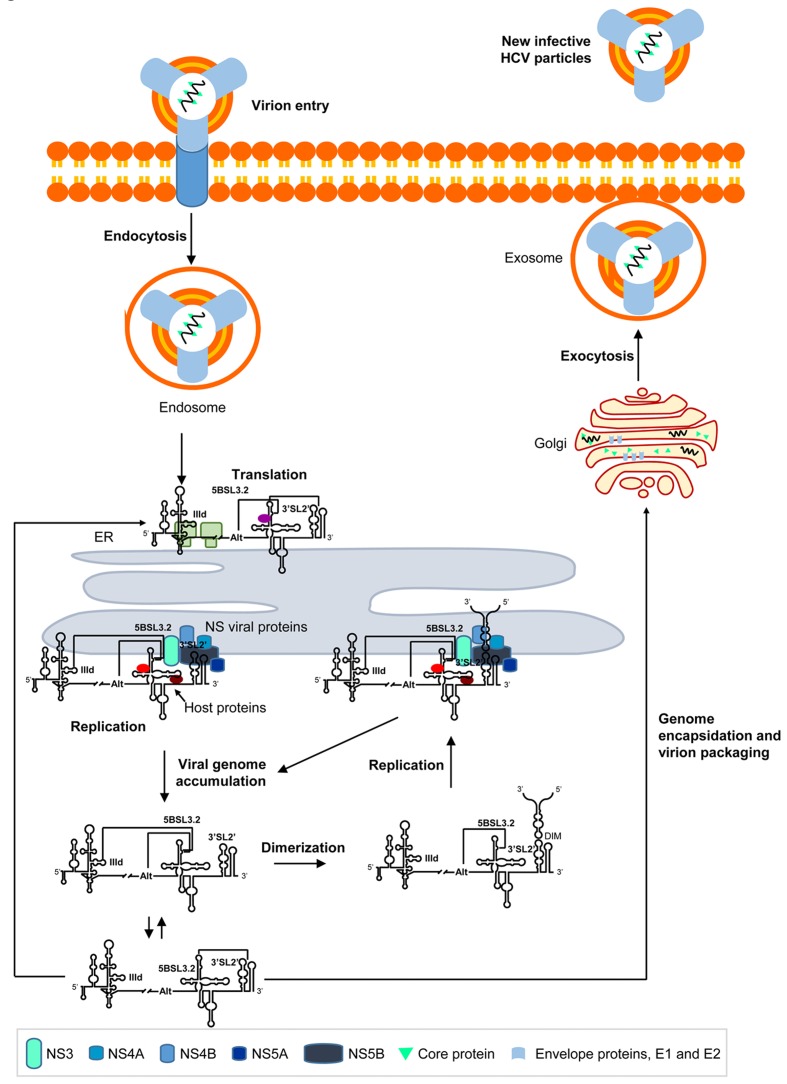
Long-range RNA–RNA interactions regulate the different steps in the HCV infective cycle. The figure shows a working model demonstrating the long-range RNA–RNA interactions in the HCV genome described to date, and their role in the progression of the infective cycle. Briefly, during early infection, the viral genome is released to the cytosol and viral translation initiates in an IRES-dependent manner on the surface of the endoplasmic reticulum (ER). Subdomain IIId is then occupied by the 40S ribosomal subunit, which impedes the interaction IIId-5BSL3.2 and enhances the conformational rearrangement at the 3′ end mediated by the 5BSL3.2 domain. The accumulation of NS viral proteins induces the formation of replication complexes, which preferentially recruit viral genomes showing the interaction IIId-5BSL3.2 or Alt-5BSL3.2. This contact favors a translationally repressed-state and enhanced replication dependent on the interaction Alt-5BSL3.2. It also interferes with the formation of dimeric genomic particles. The accumulation of newly synthesized HCV RNA genomes induces the initiation of new rounds of translation. The dimerization process is thermodynamically favored under these conditions, and the dimeric genomes produced offer optimal templates for viral replication. Alternatively, a fraction of the HCV RNA molecules is encapsidated and released to the extracellular medium by exocytosis.

During early infection, HCV virions are endocytosed and their genomes released into the cytosol by a little-understood mechanism (**Figure 6**; for a review see Scheel and Rice, 2013). Endoplasmic reticulum (ER)-associated translation is then initiated by the IRES region. During this stage, subdomain IIId of the IRES is preferentially occupied by the translational machinery, thus favoring the contacts 5BSL3.2-Alt and 5BSL3.2-3′X (**Figure 6**). The emerging HCV polyprotein is co- and post-translationally cleaved by cellular proteases and viral NS2-NS3 and NS3-NS4A proteases to release 10 HCV proteins (Grakoui et al., 1993). The accumulation of non-structural viral proteins promotes significant rearrangements in the ER membrane to create optimized microenvironments for RNA replication (as seen for other positive-strand RNA viruses) (Egger et al., 2002; Moradpour et al., 2003; Meyers et al., 2016; Falcon et al., 2017). The binding of the replicase complex to the 3′X tail of the viral genome, and the concomitant recruitment of cellular components at the CRE region (Oakland et al., 2013; Ríos-Marco et al., 2016), releases the 5BSL3.2 domain for new interactions with distant genomic RNA elements such as subdomain IIId within the IRES (**Figure 6**). This contact promotes the viral genome’s acquisition of a circular form. In addition, the interaction IIId-5BSL3.2 blocks the recruitment of 40S ribosomal particles by the IRES, contributing to the creation of a translationally repressed-state(Romero-López and Berzal-Herranz, 2012). 5BSL3.2 is thus considered a specific inhibitor of HCV IRES function (Romero-López and Berzal-Herranz, 2012). The IIId-5BSL3.2 interaction also controls the structural switch at the 3′X tail and partially interferes with the formation of dimeric genomes (Romero-López et al., 2017). From a thermodynamic point of view, the interaction IIId-5BSL3.2 can swap with the contact Alt-5BSL3.2 (Shetty et al., 2013) to create a favorable replicative environment (**Figure 6**) (Diviney et al., 2008; Tuplin et al., 2012). Viral RNA synthesis is then initiated using the positive genome strand as a template, yielding the complementary, negative strand, which is used for the generation of new progeny RNA genomes (for a review, see Kim and Chang, 2013). In a later stage of the cycle, viral genomes accumulate in the cytosol, where they serve as mRNAs for the production of HCV proteins, thus initiating new rounds of translation-replication (**Figure 6**). At this time, the IRES region is occluded by the translational machinery, favoring the interaction Alt-5BSL3.2. Because of the high concentration of viral RNAs, genomic dimerization is thermodynamically favored (**Figure 6**). This results in viral RNA storage, but HCV dimeric genomes are also excellent templates for the initiation of replication (Cantero-Camacho and Gallego, 2015; Masante et al., 2015; Cantero-Camacho et al., 2017). According to this model, HCV genomic dimerization may play a replication enhancing role during late infection. Finally, a fraction of the newly synthesized RNA molecules is shuttled for encapsidation and released to the extracellular medium (**Figure 6**).

The model proposed above recapitulates many of the findings made regarding the molecular biology of HCV. More importantly, it provides an overview of the critical role of long-distance RNA–RNA contacts in the progression of the infective cycle, and points to 5BSL3.2 as a multifunctional component encoded in the viral genome.

## Concluding Remarks

The contrast between the high structural conservation and sequence variability of the HCV RNA genome reflects an efficient mechanism for retaining essential functionalities in certain structural elements while favoring the acquisition of novel capabilities. Whereas many genomic domains govern essential steps in infection, the control exerted by 5BSL3.2 at multiple levels reflects the functional versatility and fine regulation that can be achieved by a single stem-loop. This domain is thus an interesting target for novel therapeutic agents. Exploring the structural dynamics of functional RNA domains offers a powerful, informative methodology for future drug design. Extending the knowledge acquired in HCV to related viruses, such as flaviviruses, will allow conformational maps of common structural domains to be produced, help reveal similar viral mechanisms involved in the infective cycle, and perhaps contribute to the development of novel therapeutic and diagnostic strategies.

## Author Contributions

CR-L and AB-H designed and wrote the paper.

## Conflict of Interest Statement

The authors declare that the research was conducted in the absence of any commercial or financial relationships that could be construed as a potential conflict of interest.

## References

[B1] Angulo J. Ulryck N. Deforges J. Chamond N. Lopez-Lastra M. Masquida B. (2016). LOOP IIId of the HCV IRES is essential for the structural rearrangement of the 40S-HCV IRES complex. *Nucleic Acids Res.* 44 1309–1325. 10.1093/nar/gkv1325 26626152 PMC4756818

[B2] Ariumi Y. Kuroki M. Abe K. Dansako H. Ikeda M. Wakita T. (2007). DDX3 DEAD-box RNA helicase is required for hepatitis C virus RNA replication. *J. Virol.* 81 13922–13926. 10.1128/JVI.01517-07 17855521 PMC2168844

[B3] Babaylova E. Graifer D. Malygin A. Stahl J. Shatsky I. Karpova G. (2009). Positioning of subdomain IIId and apical loop of domain II of the hepatitis C IRES on the human 40S ribosome. *Nucleic Acids Res.* 37 1141–1151. 10.1093/nar/gkn1026 19129232 PMC2651777

[B4] Bai Y. Zhou K. Doudna J. A. (2013). Hepatitis C virus 3′UTR regulates viral translation through direct interactions with the host translation machinery. *Nucleic Acids Res.* 41 7861–7874. 10.1093/nar/gkt543 23783572 PMC3763534

[B5] Bartenschlager R. Lohmann V. (2000). Replication of hepatitis C virus. *J. Gen. Virol.* 81 1631–1648. 10.1099/0022-1317-81-7-1631 10859368

[B6] Berry K. E. Waghray S. Doudna J. A. (2010). The HCV IRES pseudoknot positions the initiation codon on the 40S ribosomal subunit. *RNA* 16 1559–1569. 10.1261/rna.2197210 20584896 PMC2905755

[B7] Berry K. E. Waghray S. Mortimer S. A. Bai Y. Doudna J. A. (2011). Crystal structure of the HCV IRES central domain reveals strategy for start-codon positioning. *Structure* 19 1456–1466. 10.1016/j.str.2011.08.002 22000514 PMC3209822

[B8] Blight K. J. Rice C. M. (1997). Secondary structure determination of the conserved 98-base sequence at the 3′ terminus of hepatitis C virus genome RNA. *J. Virol.* 71 7345–7352. 9311812 10.1128/jvi.71.10.7345-7352.1997 PMC192079

[B9] Bourliere M. Gordon S. C. Flamm S. L. Cooper C. L. Ramji A. Tong M. (2017). Sofosbuvir, Velpatasvir, and Voxilaprevir for previously treated HCV infection. *N. Engl. J. Med.* 376 2134–2146. 10.1056/NEJMoa1613512 28564569

[B10] Bradrick S. S. Walters R. W. Gromeier M. (2006). The hepatitis C virus 3′-untranslated region or a poly(A) tract promote efficient translation subsequent to the initiation phase. *Nucleic Acids Res.* 34 1293–1303. 10.1093/nar/gkl019 16510853 PMC1388098

[B11] Bruscella P. Bottini S. Baudesson C. Pawlotsky J. M. Feray C. Trabucchi M. (2017). Viruses and miRNAs: more friends than foes. *Front. Microbiol.* 8:824. 10.3389/fmicb.2017.00824 28555130 PMC5430039

[B12] Bukh J. Purcell R. H. Miller R. H. (1992). Sequence analysis of the 5′ noncoding region of hepatitis C virus. *Proc. Natl. Acad. Sci. U.S.A.* 89 4942–4946. 10.1073/pnas.89.11.4942 1317578 PMC49204

[B13] Bung C. Bochkaeva Z. Terenin I. Zinovkin R. Shatsky I. N. Niepmann M. (2010). Influence of the hepatitis C virus 3′-untranslated region on IRES-dependent and cap-dependent translation initiation. *FEBS Lett.* 584 837–842. 10.1016/j.febslet.2010.01.015 20079737

[B14] Cantero-Camacho A. Fan L. Wang Y. X. Gallego J. (2017). Three-dimensional structure of the 3′X-tail of hepatitis C virus RNA in monomeric and dimeric states. *RNA* 23 1465–1476. 10.1261/rna.060632.117 28630140 PMC5558915

[B15] Cantero-Camacho A. Gallego J. (2015). The conserved 3′X terminal domain of hepatitis C virus genomic RNA forms a two-stem structure that promotes viral RNA dimerization. *Nucleic Acids Res.* 43 8529–8539. 10.1093/nar/gkv786 26240378 PMC4787799

[B16] Carter W. Connelly S. Struble K. (2017). Reinventing HCV treatment: past and future perspectives. *J. Clin. Pharmacol.* 57 287–296. 10.1002/jcph.830 27654843

[B17] Cheng J. C. Yeh Y. J. Tseng C. P. Hsu S. D. Chang Y. L. Sakamoto N. (2012). Let-7b is a novel regulator of hepatitis C virus replication. *Cell Mol. Life Sci.* 69 2621–2633. 10.1007/s00018-012-0940-6 22391672 PMC11115169

[B18] Choo Q. L. Kuo G. Weiner A. J. Overby L. R. Bradley D. W. Houghton M. (1989). Isolation of a cDNA clone derived from a blood-borne non-A, non-B viral hepatitis genome. *Science* 244 359–362. 10.1126/science.2523562 2523562

[B19] Chu D. Ren S. Hu S. Wang W. G. Subramanian A. Contreras D. (2013). Systematic analysis of enhancer and critical *cis*-acting RNA elements in the protein-encoding region of the hepatitis C virus genome. *J. Virol.* 87 5678–5696. 10.1128/JVI.00840-12 23487449 PMC3648135

[B20] Collier A. J. Gallego J. Klinck R. Cole P. T. Harris S. J. Harrison G. P. (2002). A conserved RNA structure within the HCV IRES eIF3-binding site. *Nat. Struct. Biol.* 9 375–380. 10.1038/nsb785 11927954

[B21] Cristofari G. Ivanyi-Nagy R. Gabus C. Boulant S. Lavergne J. P. Penin F. (2004). The hepatitis C virus core protein is a potent nucleic acid chaperone that directs dimerization of the viral (+) strand RNA *in vitro*. *Nucleic Acids Res.* 32 2623–2631. 10.1093/nar/gkh579 15141033 PMC419467

[B22] Cuevas J. M. Gonzalez-Candelas F. Moya A. Sanjuan R. (2009). Effect of ribavirin on the mutation rate and spectrum of hepatitis C virus in vivo. *J. Virol.* 83 5760–5764. 10.1128/JVI.00201-09 19321623 PMC2681971

[B23] Davis M. Sagan S. M. Pezacki J. P. Evans D. J. Simmonds P. (2008). Bioinformatic and physical characterizations of genome-scale ordered RNA structure in mammalian RNA viruses. *J. Virol.* 82 11824–11836. 10.1128/JVI.01078-08 18799591 PMC2583674

[B24] Diviney S. Tuplin A. Struthers M. Armstrong V. Elliott R. M. Simmonds P. (2008). A hepatitis C virus *cis*-acting replication element forms a long-range RNA-RNA interaction with upstream RNA sequences in NS5B. *J. Virol.* 82 9008–9022. 10.1128/JVI.02326-07 18614633 PMC2546899

[B25] Dmitriev S. E. Terenin I. M. Andreev D. E. Ivanov P. A. Dunaevsky J. E. Merrick W. C. (2010). GTP-independent tRNA delivery to the ribosomal P-site by a novel eukaryotic translation factor. *J. Biol. Chem.* 285 26779–26787. 10.1074/jbc.M110.119693 20566627 PMC2930676

[B26] Dreyfuss G. Kim V. N. Kataoka N. (2002). Messenger-RNA-binding proteins and the messages they carry. *Nat. Rev. Mol. Cell Biol.* 3 195–205. 10.1038/nrm760 11994740

[B27] Egger D. Wolk B. Gosert R. Bianchi L. Blum H. E. Moradpour D. (2002). Expression of hepatitis C virus proteins induces distinct membrane alterations including a candidate viral replication complex. *J. Virol.* 76 5974–5984. 10.1128/JVI.76.12.5974-5984.2002 12021330 PMC136238

[B28] Falcon V. Acosta-Rivero N. Gonzalez S. Duenas-Carrera S. Martinez-Donato G. Menendez I. (2017). Ultrastructural and biochemical basis for hepatitis C virus morphogenesis. *Virus Genes* 53 151–164. 10.1007/s11262-017-1426-2 28233195

[B29] Filbin M. E. Kieft J. S. (2011). HCV IRES domain IIb affects the configuration of coding RNA in the 40S subunit’s decoding groove. *RNA* 17 1258–1273. 10.1261/rna.2594011 21606179 PMC3138563

[B30] Filbin M. E. Vollmar B. S. Shi D. Gonen T. Kieft J. S. (2013). HCV IRES manipulates the ribosome to promote the switch from translation initiation to elongation. *Nat. Struct. Mol. Biol.* 20 150–158. 10.1038/nsmb.2465 23262488 PMC3864654

[B31] Fricke M. Dunnes N. Zayas M. Bartenschlager R. Niepmann M. Marz M. (2015). Conserved RNA secondary structures and long-range interactions in hepatitis C viruses. *RNA* 21 1219–1232. 10.1261/rna.049338.114 25964384 PMC4478341

[B32] Friebe P. Bartenschlager R. (2002). Genetic analysis of sequences in the 3′ nontranslated region of hepatitis C virus that are important for RNA replication. *J. Virol.* 76 5326–5338. 10.1128/JVI.76.11.5326-5338.2002 11991961 PMC137049

[B33] Friebe P. Boudet J. Simorre J. P. Bartenschlager R. (2005). Kissing-loop interaction in the 3′ end of the hepatitis C virus genome essential for RNA replication. *J. Virol.* 79 380–392. 10.1128/JVI.79.1.380-392.2005 15596831 PMC538730

[B34] Galli A. Bukh J. (2014). Comparative analysis of the molecular mechanisms of recombination in hepatitis C virus. *Trends Microbiol.* 22 354–364. 10.1016/j.tim.2014.02.005 24636243

[B35] Grakoui A. Wychowski C. Lin C. Feinstone S. M. Rice C. M. (1993). Expression and identification of hepatitis C virus polyprotein cleavage products. *J. Virol.* 67 1385–1395. 7679746 10.1128/jvi.67.3.1385-1395.1993 PMC237508

[B36] Guo R. Li Y. Ning J. Sun D. Lin L. Liu X. (2013). HnRNP A1/A2 and SF2/ASF regulate alternative splicing of interferon regulatory factor-3 and affect immunomodulatory functions in human non-small cell lung cancer cells. *PLOS ONE* 8:e62729. 10.1371/journal.pone.0062729 23658645 PMC3639176

[B37] Gwack Y. Kim D. W. Han J. H. Choe J. (1996). Characterization of RNA binding activity and RNA helicase activity of the hepatitis C virus NS3 protein. *Biochem. Biophys. Res. Commun.* 225 654–659. 10.1006/bbrc.1996.1225 8753814

[B38] Hashem Y. Des Georges A. Dhote V. Langlois R. Liao H. Y. Grassucci R. A. (2013). Hepatitis-C-virus-like internal ribosome entry sites displace eIF3 to gain access to the 40S subunit. *Nature* 503 539–543. 10.1038/nature12658 24185006 PMC4106463

[B39] Hofacker I. L. (2004). RNA secondary structure analysis using the Vienna RNA package. *Curr. Protoc. Bioinformatics* 26:12.2.1–12.2.16. 10.1002/0471250953.bi1202s04 19496057

[B40] Hofacker I. L. Fekete M. Flamm C. Huynen M. A. Rauscher S. Stolorz P. E. (1998). Automatic detection of conserved RNA structure elements in complete RNA virus genomes. *Nucleic Acids Res.* 26 3825–3836. 10.1093/nar/26.16.3825 9685502 PMC147758

[B41] Honda M. Brown E. A. Lemon S. M. (1996a). Stability of a stem-loop involving the initiator AUG controls the efficiency of internal initiation of translation on hepatitis C virus RNA. *RNA* 2 955–968. 8849773 PMC1369429

[B42] Honda M. Ping L. H. Rijnbrand R. C. Amphlett E. Clarke B. Rowlands D. (1996b). Structural requirements for initiation of translation by internal ribosome entry within genome-length hepatitis C virus RNA. *Virology* 222 31–42. 8806485 10.1006/viro.1996.0395

[B43] Hoofnagle J. H. (2002). Course and outcome of hepatitis C. *Hepatology* 36 S21–S29. 10.1053/jhep.2002.36227 12407573

[B44] Huang L. Hwang J. Sharma S. D. Hargittai M. R. Chen Y. Arnold J. J. (2005). Hepatitis C virus non-structural protein 5A (NS5A) is a RNA-binding protein. *J. Biol. Chem.* 280 36417–36428. 10.1074/jbc.M508175200 16126720

[B45] Irshad M. Ansari M. A. Singh A. Nag P. Raghvendra L. Singh S. (2010). HCV-genotypes: a review on their origin, global status, assay system, pathogenecity and response to treatment. *Hepatogastroenterology* 57 1529–1538. 21443116

[B46] Ito T. Lai M. M. (1997). Determination of the secondary structure of and cellular protein binding to the 3′-untranslated region of the hepatitis C virus RNA genome. *J. Virol.* 71 8698–8706. 9343228 10.1128/jvi.71.11.8698-8706.1997 PMC192334

[B47] Ivanyi-Nagy R. Kanevsky I. Gabus C. Lavergne J. P. Ficheux D. Penin F. (2006). Analysis of hepatitis C virus RNA dimerization and core-RNA interactions. *Nucleic Acids Res.* 34 2618–2633. 10.1093/nar/gkl240 16707664 PMC1463901

[B48] Jaafar Z. A. Oguro A. Nakamura Y. Kieft J. S. (2016). Translation initiation by the hepatitis C virus IRES requires eIF1A and ribosomal complex remodeling. *Elife* 5:e21198. 10.7554/eLife.21198 28009256 PMC5238962

[B49] Jangra R. K. Yi M. Lemon S. M. (2010). Regulation of hepatitis C virus translation and infectious virus production by the microRNA miR-122. *J. Virol.* 84 6615–6625. 10.1128/JVI.00417-10 20427538 PMC2903297

[B50] Jopling C. L. Yi M. Lancaster A. M. Lemon S. M. Sarnow P. (2005). Modulation of hepatitis C virus RNA abundance by a liver-specific microRNA. *Science* 309 1577–1581. 10.1126/science.1113329 16141076

[B51] Jubin R. Vantuno N. E. Kieft J. S. Murray M. G. Doudna J. A. Lau J. Y. (2000). Hepatitis C virus internal ribosome entry site (IRES) stem loop IIId contains a phylogenetically conserved GGG triplet essential for translation and IRES folding. *J. Virol.* 74 10430–10437. 10.1128/JVI.74.22.10430-10437.2000 11044087 PMC110917

[B52] Jung J. H. Park J. H. Jee M. H. Keum S. J. Cho M. S. Yoon S. K. (2011). Hepatitis C virus infection is blocked by HMGB1 released from virus-infected cells. *J. Virol.* 85 9359–9368. 10.1128/JVI.00682-11 21752923 PMC3165778

[B53] Kao C. C. Yang X. Kline A. Wang Q. M. Barket D. Heinz B. A. (2000). Template requirements for RNA synthesis by a recombinant hepatitis C virus RNA-dependent RNA polymerase. *J. Virol.* 74 11121–11128. 10.1128/JVI.74.23.11121-11128.2000 11070008 PMC113194

[B54] Kieft J. S. Zhou K. Jubin R. Murray M. G. Lau J. Y. Doudna J. A. (1999). The hepatitis C virus internal ribosome entry site adopts an ion-dependent tertiary fold. *J. Mol. Biol.* 292 513–529. 10.1006/jmbi.1999.3095 10497018

[B55] Kim C. W. Chang K. M. (2013). Hepatitis C virus: virology and life cycle. *Clin. Mol. Hepatol.* 19 17–25. 10.3350/cmh.2013.19.1.17 23593605 PMC3622851

[B56] Kim J. H. Park S. M. Park J. H. Keum S. J. Jang S. K. (2011). eIF2A mediates translation of hepatitis C viral mRNA under stress conditions. *EMBO J.* 30 2454–2464. 10.1038/emboj.2011.146 21556050 PMC3116280

[B57] Kolupaeva V. G. Pestova T. V. Hellen C. U. (2000). An enzymatic footprinting analysis of the interaction of 40S ribosomal subunits with the internal ribosomal entry site of hepatitis C virus. *J. Virol.* 74 6242–6250. 10.1128/JVI.74.14.6242-6250.2000 10864633 PMC112129

[B58] Kolykhalov A. A. Feinstone S. M. Rice C. M. (1996). Identification of a highly conserved sequence element at the 3′ terminus of hepatitis C virus genome RNA. *J. Virol.* 70 3363–3371. 8648666 10.1128/jvi.70.6.3363-3371.1996 PMC190207

[B59] Kolykhalov A. A. Mihalik K. Feinstone S. M. Rice C. M. (2000). Hepatitis C virus-encoded enzymatic activities and conserved RNA elements in the 3′ nontranslated region are essential for virus replication in vivo. *J. Virol.* 74 2046–2051. 10.1128/JVI.74.4.2046-2051.2000 10644379 PMC111684

[B60] Kowdley K. V. Lawitz E. Crespo I. Hassanein T. Davis M. N. Demicco M. (2013). Sofosbuvir with pegylated interferon alfa-2a and ribavirin for treatment-naive patients with hepatitis C genotype-1 infection (ATOMIC): an open-label, randomised, multicentre phase 2 trial. *Lancet* 381 2100–2107. 10.1016/S0140-6736(13)60247-0 23499440

[B61] Kranawetter C. Brady S. Sun L. Schroeder M. Chen S. J. Heng X. (2017). Nuclear magnetic resonance study of RNA structures at the 3′-end of the hepatitis C virus genome. *Biochemistry* 56 4972–4984. 10.1021/acs.biochem.7b00573 28829576 PMC5643200

[B62] Lawitz E. Lalezari J. P. Hassanein T. Kowdley K. V. Poordad F. F. Sheikh A. M. (2013). Sofosbuvir in combination with peginterferon alfa-2a and ribavirin for non-cirrhotic, treatment-naive patients with genotypes 1, 2, and 3 hepatitis C infection: a randomised, double-blind, phase 2 trial. *Lancet Infect. Dis.* 13 401–408. 10.1016/S1473-3099(13)70033-1 23499158

[B63] Le Guillou-Guillemette H. Vallet S. Gaudy-Graffin C. Payan C. Pivert A. Goudeau A. (2007). Genetic diversity of the hepatitis C virus: impact and issues in the antiviral therapy. *World J. Gastroenterol.* 13 2416–2426. 10.3748/wjg.v13.i17.2416 17552024 PMC4146759

[B64] Lee H. Shin H. Wimmer E. Paul A. V. (2004). *cis*-acting RNA signals in the NS5B C-terminal coding sequence of the hepatitis C virus genome. *J. Virol.* 78 10865–10877. 10.1128/JVI.78.20.10865-10877.2004 15452207 PMC521798

[B65] Li K. Lemon S. M. (2013). Innate immune responses in hepatitis C virus infection. *Semin. Immunopathol.* 35 53–72. 10.1007/s00281-012-0332-x 22868377 PMC3732459

[B66] Lohmann V. Korner F. Herian U. Bartenschlager R. (1997). Biochemical properties of hepatitis C virus NS5B RNA-dependent RNA polymerase and identification of amino acid sequence motifs essential for enzymatic activity. *J. Virol.* 71 8416–8428. 9343198 10.1128/jvi.71.11.8416-8428.1997 PMC192304

[B67] Lourenco S. Costa F. Debarges B. Andrieu T. Cahour A. (2008). Hepatitis C virus internal ribosome entry site-mediated translation is stimulated by *cis*-acting RNA elements and trans-acting viral factors. *FEBS J.* 275 4179–4197. 10.1111/j.1742-4658.2008.06566.x 18637118

[B68] Lu H. Li W. Noble W. S. Payan D. Anderson D. C. (2004). Riboproteomics of the hepatitis C virus internal ribosomal entry site. *J. Proteome Res.* 3 949–957. 10.1021/pr0499592 15473682

[B69] Lukavsky P. J. Otto G. A. Lancaster A. M. Sarnow P. Puglisi J. D. (2000). Structures of two RNA domains essential for hepatitis C virus internal ribosome entry site function. *Nat. Struct. Biol.* 7 1105–1110. 10.1038/81951 11101890

[B70] Lytle J. R. Wu L. Robertson H. D. (2002). Domains on the hepatitis C virus internal ribosome entry site for 40s subunit binding. *RNA* 8 1045–1055. 10.1017/S1355838202029965 12212848 PMC1370315

[B71] Malygin A. A. Kossinova O. A. Shatsky I. N. Karpova G. G. (2013a). HCV IRES interacts with the 18S rRNA to activate the 40S ribosome for subsequent steps of translation initiation. *Nucleic Acids Res.* 41 8706–8714. 10.1093/nar/gkt632 23873958 PMC3794592

[B72] Malygin A. A. Shatsky I. N. Karpova G. G. (2013b). Proteins of the human 40S ribosomal subunit involved in hepatitis C IRES binding as revealed from fluorescent labeling. *Biochemistry* 78 53–59. 10.1134/S0006297913010069 23379559

[B73] Manrubia S. Lazaro E. (2016). Getting to know viral evolutionary strategies: towards the next generation of quasispecies models. *Curr. Top. Microbiol. Immunol.* 392 201–217. 10.1007/82_2015_457 26271604

[B74] Markoff L. (2003). 5′- and 3′-noncoding regions in flavivirus RNA. *Adv. Virus Res.* 59 177–228. 10.1016/S0065-3527(03)59006-6 14696330 PMC7119107

[B75] Martell M. Esteban J. I. Quer J. Genesca J. Weiner A. Esteban R. (1992). Hepatitis C virus (HCV) circulates as a population of different but closely related genomes: quasispecies nature of HCV genome distribution. *J. Virol.* 66 3225–3229. 1313927 10.1128/jvi.66.5.3225-3229.1992 PMC241092

[B76] Masante C. Jaubert C. Palau W. Plissonneau J. Besnard L. Ventura M. (2015). Mutations of the SL2 dimerization sequence of the hepatitis C genome abrogate viral replication. *Cell Mol. Life Sci.* 72 3375–3385. 10.1007/s00018-015-1893-3 25822205 PMC7079775

[B77] Matsuda D. Mauro V. P. (2014). Base pairing between hepatitis C virus RNA and 18S rRNA is required for IRES-dependent translation initiation *in vivo*. *Proc. Natl. Acad. Sci. U.S.A.* 111 15385–15389. 10.1073/pnas.1413472111 25313046 PMC4217472

[B78] Mauger D. M. Golden M. Yamane D. Williford S. Lemon S. M. Martin D. P. (2015). Functionally conserved architecture of hepatitis C virus RNA genomes. *Proc. Natl. Acad. Sci. U.S.A.* 112 3692–3697. 10.1073/pnas.1416266112 25775547 PMC4378395

[B79] Mccaffrey A. P. Ohashi K. Meuse L. Shen S. Lancaster A. M. Lukavsky P. J. (2002). Determinants of hepatitis C translational initiation *in vitro*, in cultured cells and mice. *Mol. Ther.* 5 676–684. 10.1006/mthe.2002.0600 12027551

[B80] Mcmullan L. K. Grakoui A. Evans M. J. Mihalik K. Puig M. Branch A. D. (2007). Evidence for a functional RNA element in the hepatitis C virus core gene. *Proc. Natl. Acad. Sci. U.S.A.* 104 2879–2884. 10.1073/pnas.0611267104 17299041 PMC1815275

[B81] Messina J. P. Humphreys I. Flaxman A. Brown A. Cooke G. S. Pybus O. G. (2015). Global distribution and prevalence of hepatitis C virus genotypes. *Hepatology* 61 77–87. 10.1002/hep.27259 25069599 PMC4303918

[B82] Meyers N. L. Fontaine K. A. Kumar G. R. Ott M. (2016). Entangled in a membranous web: ER and lipid droplet reorganization during hepatitis C virus infection. *Curr. Opin. Cell Biol.* 41 117–124. 10.1016/j.ceb.2016.05.003 27240021 PMC5477849

[B83] Mohd Hanafiah K. Groeger J. Flaxman A. D. Wiersma S. T. (2013). Global epidemiology of hepatitis C virus infection: new estimates of age-specific antibody to HCV seroprevalence. *Hepatology* 57 1333–1342. 10.1002/hep.26141 23172780

[B84] Moradpour D. Gosert R. Egger D. Penin F. Blum H. E. Bienz K. (2003). Membrane association of hepatitis C virus nonstructural proteins and identification of the membrane alteration that harbors the viral replication complex. *Antiviral Res.* 60 103–109. 10.1016/j.antiviral.2003.08.017 14638405

[B85] Morel V. Fournier C. Francois C. Brochot E. Helle F. Duverlie G. (2011). Genetic recombination of the hepatitis C virus: clinical implications. *J. Viral. Hepat.* 18 77–83. 10.1111/j.1365-2893.2010.01367.x 21235686

[B86] Murakami Y. Aly H. H. Tajima A. Inoue I. Shimotohno K. (2009). Regulation of the hepatitis C virus genome replication by miR-199a. *J. Hepatol.* 50 453–460. 10.1016/j.jhep.2008.06.010 19144437

[B87] Oakland T. E. Haselton K. J. Randall G. (2013). EWSR1 binds the hepatitis C virus *cis*-acting replication element and is required for efficient viral replication. *J. Virol.* 87 6625–6634. 10.1128/JVI.01006-12 23552423 PMC3676111

[B88] Otto G. A. Lukavsky P. J. Lancaster A. M. Sarnow P. Puglisi J. D. (2002). Ribosomal proteins mediate the hepatitis C virus IRES-HeLa 40S interaction. *RNA* 8 913–923. 10.1017/S1355838202022057 12166646 PMC1370308

[B89] Palau W. Masante C. Ventura M. Di Primo C. (2013). Direct evidence for RNA-RNA interactions at the 3′ end of the Hepatitis C virus genome using surface plasmon resonance. *RNA* 19 982–991. 10.1261/rna.037606.112 23651615 PMC3683932

[B90] Paronetto M. P. Minana B. Valcarcel J. (2011). The Ewing sarcoma protein regulates DNA damage-induced alternative splicing. *Mol. Cell* 43 353–368. 10.1016/j.molcel.2011.05.035 21816343

[B91] Patino-Galindo J. A. Gonzalez-Candelas F. (2017). Comparative analysis of variation and selection in the HCV genome. *Infect. Genet. Evol.* 49 104–110. 10.1016/j.meegid.2017.01.010 28087495

[B92] Perales C. Domingo E. (2016). Antiviral strategies based on lethal mutagenesis and error threshold. *Curr. Top. Microbiol. Immunol.* 392 323–339. 10.1007/82_2015_459 26294225

[B93] Perard J. Leyrat C. Baudin F. Drouet E. Jamin M. (2013). Structure of the full-length HCV IRES in solution. *Nat. Commun.* 4:1612. 10.1038/ncomms2611 23511476

[B94] Pestova T. V. Shatsky I. N. Fletcher S. P. Jackson R. J. Hellen C. U. (1998). A prokaryotic-like mode of cytoplasmic eukaryotic ribosome binding to the initiation codon during internal translation initiation of hepatitis C and classical swine fever virus RNAs. *Genes Dev.* 12 67–83. 10.1101/gad.12.1.67 9420332 PMC316404

[B95] Pirakitikulr N. Kohlway A. Lindenbach B. D. Pyle A. M. (2016). The coding region of the HCV genome contains a network of regulatory RNA structures. *Mol. Cell* 62 111–120. 10.1016/j.molcel.2016.01.024 26924328 PMC4826301

[B96] Quade N. Boehringer D. Leibundgut M. Van Den Heuvel J. Ban N. (2015). Cryo-EM structure of hepatitis C virus IRES bound to the human ribosome at 3.9-A resolution. *Nat. Commun.* 6:7646. 10.1038/ncomms8646 26155016 PMC4510694

[B97] Randall G. Panis M. Cooper J. D. Tellinghuisen T. L. Sukhodolets K. E. Pfeffer S. (2007). Cellular cofactors affecting hepatitis C virus infection and replication. *Proc. Natl. Acad. Sci. U.S.A.* 104 12884–12889. 10.1073/pnas.0704894104 17616579 PMC1937561

[B98] Reynolds J. E. Kaminski A. Kettinen H. J. Grace K. Clarke B. E. Carroll A. R. (1995). Unique features of internal initiation of hepatitis C virus RNA translation. *EMBO J.* 14 6010–6020. 8846793 10.1002/j.1460-2075.1995.tb00289.x PMC394721

[B99] Ribeiro R. M. Li H. Wang S. Stoddard M. B. Learn G. H. Korber B. T. (2012). Quantifying the diversification of hepatitis C virus (HCV) during primary infection: estimates of the *in vivo* mutation rate. *PLOS Pathog.* 8:e1002881. 10.1371/journal.ppat.1002881 22927817 PMC3426522

[B100] Ríos-Marco P. Romero-López C. Berzal-Herranz A. (2016). The *cis*-acting replication element of the hepatitis C virus genome recruits host factors that influence viral replication and translation. *Sci. Rep.* 6:25729. 10.1038/srep25729 27165399 PMC4863150

[B101] Roberts A. P. Lewis A. P. Jopling C. L. (2011). miR-122 activates hepatitis C virus translation by a specialized mechanism requiring particular RNA components. *Nucleic Acids Res.* 39 7716–7729. 10.1093/nar/gkr426 21653556 PMC3177192

[B102] Romero-López C. Barroso-delJesus A. Berzal-Herranz A. (2017). The chaperone-like activity of the hepatitis C virus IRES and CRE elements regulates genome dimerization. *Sci. Rep.* 7:43415. 10.1038/srep43415 28233845 PMC5324077

[B103] Romero-López C. Barroso-delJesus A. García-Sacristán A. Briones C. Berzal-Herranz A. (2012). The folding of the hepatitis C virus internal ribosome entry site depends on the 3′-end of the viral genome. *Nucleic Acids Res.* 40 11697–11713. 10.1093/nar/gks927 23066110 PMC3526292

[B104] Romero-López C. Barroso-delJesus A. García-Sacristán A. Briones C. Berzal-Herranz A. (2014). End-to-end crosstalk within the hepatitis C virus genome mediates the conformational switch of the 3′X-tail region. *Nucleic Acids Res.* 42 567–582. 10.1093/nar/gkt841 24049069 PMC3874160

[B105] Romero-López C. Berzal-Herranz A. (2009). A long-range RNA-RNA interaction between the 5′ and 3′ ends of the HCV genome. *RNA* 15 1740–1752. 10.1261/rna.1680809 19605533 PMC2743058

[B106] Romero-López C. Berzal-Herranz A. (2012). The functional RNA domain 5BSL3.2 within the NS5B coding sequence influences hepatitis C virus IRES-mediated translation. *Cell Mol. Life Sci.* 69 103–113. 10.1007/s00018-011-0729-z 21598019 PMC11115049

[B107] Romero-López C. Berzal-Herranz A. (2013). Unmasking the information encoded as structural motifs of viral RNA genomes: a potential antiviral target. *Rev. Med. Virol.* 23 340–354. 10.1002/rmv.1756 23983005 PMC7169113

[B108] Romero-López C. Berzal-Herranz A. (2015). Current and emerging themes in the structural analysis of viral RNA genomes: applications for the development of novel therapeutic drugs. *Genom. Comp. Biol.* 1:e15 10.18547/gcb.2015.vol1.iss1.e15

[B109] Scheel T. K. Galli A. Li Y. P. Mikkelsen L. S. Gottwein J. M. Bukh J. (2013). Productive homologous and non-homologous recombination of hepatitis C virus in cell culture. *PLOS Pathog.* 9:e1003228. 10.1371/journal.ppat.1003228 23555245 PMC3610614

[B110] Scheel T. K. Rice C. M. (2013). Understanding the hepatitis C virus life cycle paves the way for highly effective therapies. *Nat. Med.* 19 837–849. 10.1038/nm.3248 23836234 PMC3984536

[B111] Shetty S. Kim S. Shimakami T. Lemon S. M. Mihailescu M. R. (2010). Hepatitis C virus genomic RNA dimerization is mediated via a kissing complex intermediate. *RNA* 16 913–925. 10.1261/rna.1960410 20360391 PMC2856886

[B112] Shetty S. Stefanovic S. Mihailescu M. R. (2013). Hepatitis C virus RNA: molecular switches mediated by long-range RNA-RNA interactions? *Nucleic Acids Res.* 41 2526–2540. 10.1093/nar/gks1318 23275555 PMC3575821

[B113] Shulla A. Randall G. (2015). Spatiotemporal analysis of hepatitis C virus infection. *PLOS Pathog.* 11:e1004758. 10.1371/journal.ppat.1004758 25822891 PMC4378894

[B114] Simmonds P. Tuplin A. Evans D. J. (2004). Detection of genome-scale ordered RNA structure (GORS) in genomes of positive-stranded RNA viruses: implications for virus evolution and host persistence. *RNA* 10 1337–1351. 10.1261/rna.7640104 15273323 PMC1370621

[B115] Sizova D. V. Kolupaeva V. G. Pestova T. V. Shatsky I. N. Hellen C. U. (1998). Specific interaction of eukaryotic translation initiation factor 3 with the 5′ nontranslated regions of hepatitis C virus and classical swine fever virus RNAs. *J. Virol.* 72 4775–4782. 9573242 10.1128/jvi.72.6.4775-4782.1998 PMC110013

[B116] Smith D. B. Bukh J. Kuiken C. Muerhoff A. S. Rice C. M. Stapleton J. T. (2014). Expanded classification of hepatitis C virus into 7 genotypes and 67 subtypes: updated criteria and genotype assignment web resource. *Hepatology* 59 318–327. 10.1002/hep.26744 24115039 PMC4063340

[B117] Smith D. B. Mellor J. Jarvis L. M. Davidson F. Kolberg J. Urdea M. (1995). Variation of the hepatitis C virus 5′ non-coding region: implications for secondary structure, virus detection and typing. The International HCV collaborative study group. *J. Gen. Virol.* 76 (Pt 7), 1749–1761. 10.1099/0022-1317-76-7-1749 9049380

[B118] Smith D. B. Simmonds P. (1997). Characteristics of nucleotide substitution in the hepatitis C virus genome: constraints on sequence change in coding regions at both ends of the genome. *J. Mol. Evol.* 45 238–246. 10.1007/PL00006226 9302317

[B119] Song Y. Friebe P. Tzima E. Junemann C. Bartenschlager R. Niepmann M. (2006). The hepatitis C virus RNA 3′-untranslated region strongly enhances translation directed by the internal ribosome entry site. *J. Virol.* 80 11579–11588. 10.1128/JVI.00675-06 16971433 PMC1642618

[B120] Spahn C. M. Kieft J. S. Grassucci R. A. Penczek P. A. Zhou K. Doudna J. A. (2001). Hepatitis C virus IRES RNA-induced changes in the conformation of the 40S ribosomal subunit. *Science* 291 1959–1962. 10.1126/science.1058409 11239155

[B121] Sun C. Querol-Audi J. Mortimer S. A. Arias-Palomo E. Doudna J. A. Nogales E. (2013). Two RNA-binding motifs in eIF3 direct HCV IRES-dependent translation. *Nucleic Acids Res.* 41 7512–7521. 10.1093/nar/gkt510 23766293 PMC3753635

[B122] Takamizawa J. Konishi H. Yanagisawa K. Tomida S. Osada H. Endoh H. (2004). Reduced expression of the let-7 microRNAs in human lung cancers in association with shortened postoperative survival. *Cancer Res.* 64 3753–3756. 10.1158/0008-5472.CAN-04-0637 15172979

[B123] Tanaka T. Kato N. Cho M. J. Shimotohno K. (1995). A novel sequence found at the 3′ terminus of hepatitis C virus genome. *Biochem. Biophys. Res. Commun.* 215 744–749. 10.1006/bbrc.1995.2526 7488017

[B124] Tanaka T. Kato N. Cho M. J. Sugiyama K. Shimotohno K. (1996). Structure of the 3′ terminus of the hepatitis C virus genome. *J. Virol.* 70 3307–3312. 8627816 10.1128/jvi.70.5.3307-3312.1996 PMC190199

[B125] Terenin I. M. Dmitriev S. E. Andreev D. E. Shatsky I. N. (2008). Eukaryotic translation initiation machinery can operate in a bacterial-like mode without eIF2. *Nat. Struct. Mol. Biol.* 15 836–841. 10.1038/nsmb.1445 18604219

[B126] Tsukiyama-Kohara K. Iizuka N. Kohara M. Nomoto A. (1992). Internal ribosome entry site within hepatitis C virus RNA. *J. Virol.* 66 1476–1483. 1310759 10.1128/jvi.66.3.1476-1483.1992 PMC240872

[B127] Tuplin A. Evans D. J. Simmonds P. (2004). Detailed mapping of RNA secondary structures in core and NS5B-encoding region sequences of hepatitis C virus by RNase cleavage and novel bioinformatic prediction methods. *J. Gen. Virol.* 85 3037–3047. 10.1099/vir.0.80141-0 15448367

[B128] Tuplin A. Struthers M. Cook J. Bentley K. Evans D. J. (2015). Inhibition of HCV translation by disrupting the structure and interactions of the viral CRE and 3′ X-tail. *Nucleic Acids Res.* 43 2914–2926. 10.1093/nar/gkv142 25712095 PMC4357731

[B129] Tuplin A. Struthers M. Simmonds P. Evans D. J. (2012). A twist in the tail: SHAPE mapping of long-range interactions and structural rearrangements of RNA elements involved in HCV replication. *Nucleic Acids Res.* 40 6908–6921. 10.1093/nar/gks370 22561372 PMC3413155

[B130] Tuplin A. Wood J. Evans D. J. Patel A. H. Simmonds P. (2002). Thermodynamic and phylogenetic prediction of RNA secondary structures in the coding region of hepatitis C virus. *RNA* 8 824–841. 10.1017/S1355838202554066 12088154 PMC1370300

[B131] Villordo S. M. Gamarnik A. V. (2009). Genome cyclization as strategy for flavivirus RNA replication. *Virus Res.* 139 230–239. 10.1016/j.virusres.2008.07.016 18703097 PMC5440119

[B132] Walewski J. L. Keller T. R. Stump D. D. Branch A. D. (2001). Evidence for a new hepatitis C virus antigen encoded in an overlapping reading frame. *RNA* 7 710–721. 10.1017/S1355838201010111 11350035 PMC1370123

[B133] Wang C. Sarnow P. Siddiqui A. (1993). Translation of human hepatitis C virus RNA in cultured cells is mediated by an internal ribosome-binding mechanism. *J. Virol.* 67 3338–3344. 8388503 10.1128/jvi.67.6.3338-3344.1993 PMC237677

[B134] Wang Y. L. Chen H. Zhan Y. Q. Yin R. H. Li C. Y. Ge C. H. (2016). EWSR1 regulates mitosis by dynamically influencing microtubule acetylation. *Cell Cycle* 15 2202–2215. 10.1080/15384101.2016.1200774 27341063 PMC4993540

[B135] Weinlich S. Huttelmaier S. Schierhorn A. Behrens S. E. Ostareck-Lederer A. Ostareck D. H. (2009). IGF2BP1 enhances HCV IRES-mediated translation initiation via the 3′UTR. *RNA* 15 1528–1542. 10.1261/rna.1578409 19541769 PMC2714754

[B136] Witteveldt J. Blundell R. Maarleveld J. J. Mcfadden N. Evans D. J. Simmonds P. (2014). The influence of viral RNA secondary structure on interactions with innate host cell defences. *Nucleic Acids Res.* 42 3314–3329. 10.1093/nar/gkt1291 24335283 PMC3950689

[B137] Yamada N. Tanihara K. Takada A. Yorihuzi T. Tsutsumi M. Shimomura H. (1996). Genetic organization and diversity of the 3′ noncoding region of the hepatitis C virus genome. *Virology* 223 255–261. 10.1006/viro.1996.0476 8806561

[B138] Yamamoto H. Collier M. Loerke J. Ismer J. Schmidt A. Hilal T. (2015). Molecular architecture of the ribosome-bound Hepatitis C Virus internal ribosomal entry site RNA. *EMBO J.* 34 3042–3058. 10.15252/embj.201592469 26604301 PMC4687786

[B139] Yamamoto H. Unbehaun A. Loerke J. Behrmann E. Collier M. Burger J. (2014). Structure of the mammalian 80S initiation complex with initiation factor 5B on HCV-IRES RNA. *Nat. Struct. Mol. Biol.* 21 721–727. 10.1038/nsmb.2859 25064512

[B140] Yi M. Lemon S. M. (2003). 3′ nontranslated RNA signals required for replication of hepatitis C virus RNA. *J. Virol.* 77 3557–3568. 10.1128/JVI.77.6.3557-3568.2003 12610131 PMC149512

[B141] You S. Rice C. M. (2008). 3′ RNA elements in hepatitis C virus replication: kissing partners and long poly(U). *J. Virol.* 82 184–195. 10.1128/JVI.01796-07 17942554 PMC2224383

[B142] You S. Stump D. D. Branch A. D. Rice C. M. (2004). A *cis*-acting replication element in the sequence encoding the NS5B RNA-dependent RNA polymerase is required for hepatitis C virus RNA replication. *J. Virol.* 78 1352–1366. 10.1128/JVI.78.3.1352-1366.2004 14722290 PMC321395

[B143] Yu F. Yao H. Zhu P. Zhang X. Pan Q. Gong C. (2007). let-7 regulates self renewal and tumorigenicity of breast cancer cells. *Cell* 131 1109–1123. 10.1016/j.cell.2007.10.054 18083101

[B144] Zhang J. Yamada O. Sakamoto T. Yoshida H. Araki H. Murata T. (2005). Inhibition of hepatitis C virus replication by pol III-directed overexpression of RNA decoys corresponding to stem-loop structures in the NS5B coding region. *Virology* 342 276–285. 10.1016/j.virol.2005.08.003 16139319

[B145] Zinszner H. Albalat R. Ron D. (1994). A novel effector domain from the RNA-binding protein TLS or EWS is required for oncogenic transformation by CHOP. *Genes Dev.* 8 2513–2526. 10.1101/gad.8.21.2513 7958914

